# Pharmacotherapy response and regional cerebral blood flow characteristics in patients with obsessive-compulsive disorder

**DOI:** 10.1186/1744-9081-9-31

**Published:** 2013-07-30

**Authors:** Sheng-lin Wen, Mu-hua Cheng, Min-feng Cheng, Ji-hui Yue, Hong Wang

**Affiliations:** 1 Department of Psychiatry, Third Affiliated Hospital, Sun Yat-sen University, GuangZhou, Guangdong Province 510630, China; 2 Department of Nuclear Medicine, Third Affiliated Hospital, Sun Yat-sen University, GuangZhou, Guangdong Province 510630, China; 3 Department of Psychology, Fifth Affiliated Hospital, Sun Yat-sen University, Zhuhai, Guangdong Province 519000, China

**Keywords:** Obsessive-compulsive disorder, Regional cerebral blood flow, SSRI, Quetiapine, Therapy response

## Abstract

**Objective:**

To analyze the correlation between the pharmacotherapy response and the characteristics of the pre-treatment regional cerebral blood flow (rCBF) in patients with obsessive-compulsive disorder (OCD).

**Methods:**

Single-photon emission-computed tomography (SPECT) was used to determine the pre-treatment rCBF in 30 OCD patients and 30 normal controls. Based on their clinical remission response, the subjects were divided into two groups: selective serotonin reuptake inhibitors (SSRIs) and SSRIs plus quetiapine. The subjects with clinical remission response were identified after treatment for a period of 24 weeks, and the rCBF imaging data were processed using statistical parametric mapping (SPM) software with two-sample Z-tests.

**Results:**

Nineteen OCD patients who achieved clinical remission were included in the study. Increased rCBF in forebrain regions, including the frontal lobe, cingulate gyrus, hypothalamus, and basal ganglia, was found in 11 responders to SSRIs compared to normal control patients. The eight SSRI plus quetiapine responders exhibited a decrease in rCBF within posterior brain regions, including the parietal lobe, cerebellar vermis, and occipital lobe, and an increase in rCBF in the frontal lobe, thalamus, basal ganglia, and cerebellum tonsil compared to normal control patients.

**Conclusions:**

The characteristics of increased rCBF in forebrain regions and decreased rCBF in posterior brain regions before treatment of OCD patients was a potentially predictor of treatment response to guide treatment options.

## Introduction

Functional imaging studies using positron emission tomography (PET), functional and structural magnetic resonance imaging (fMRI), and single-photon emission tomography (SPECT) techniques have indicated that the pathophysiology of obsessive–compulsive disorder (OCD) involves widely distributed, large-scale brain systems including the orbitofrontal cortex (OFC), the anterior cingulate cortex (ACC), the dorsolateral prefrontal cortex (DLPC), the head of the caudate nucleus, and the thalamus [1-3]. The imaging of regional cerebral blood flow (rCBF) using SPECT is also a useful tool for studying OCD, as changes in rCBF generally accompany cerebral dysfunction. Functional imaging studies using SPECT techniques have revealed both increased and decreased rCBF in various brain regions, including the basal ganglia, cingulate cortex, orbitofrontal cortex, and anterior cingulate, in patients with OCD compared to normal controls [4-6]. Some researchers suggested that the changes in spontaneous neuronal activity within posterior brain regions, including the inferior parietal cortex, occipital lobe and cerebellum, played an important role in the pathophysiology in patients with OCD [3,7]. In a SPECT study, Busatto [6] also reported that the rCBF within the cerebellum was greater in patients with OCD compared to healthy control subjects. These studies proposed that a dysfunction in forebrain and/or posterior brain regions would result in the emergence and maintenance of repetitive thoughts and characteristic OCD-like behavior.

Selective serotonin reuptake inhibitors (SSRIs) have proven effective for the treatment of OCD in a number of open and controlled trials [8,9]. Several functional imaging studies of OCD patients both before and after treatments using either SSRIs or behavioral therapy suggested that the activity in the OFC, ACC, DLPC, thalamus, and caudate nucleus was decreased by successful treatments [10-12]. Although SSRIs are effective in treating OCD patients, 40%–60% of patients do not show an adequate response to these medications [13,14].

In clinical practice, the addition of a low-dose, atypical antipsychotic such as risperidone or quetiapine to ongoing SSRI treatment has been shown to be effective [15,16]. Sumitani [17] reported that OCD patients who responded to an SSRI plus atypical antipsychotic showed distinct biological abnormalities in the anterior cingulate. Buchsbaum [18] found that the successful treatment with SSRIs plus risperidone of OCD patients who were non-respondent to serotonin reuptake inhibitors alone was associated with relatively low metabolic rates in the striatum and anterior cingulate gyrus. Only one third of treatment-refractory OCD patients show a meaningful treatment response to antipsychotic augmentation [13]. Therefore, OCD patients who respond to different pharmacotherapies would show differential changes in brain perfusion in those regions affected by the treatment; these studies suggest that the OCD is a highly heterogeneous condition. It is possible that there are biological differences among OCD patients that create this special subgroup that shows pharmacological response. On the basis of previous studies, we proposed that abnormal activation involving forebrain and posterior brain regions might affect the interference processes that are associated with the pathophysiology of OCD, and that a difference in pre-treatment rCBF might predict the pharmacotherapy response to different treatments.

To understand the underlying pathophysiology better and analyze the correlation between the pharmacotherapy response and the characteristics of the pre-treatment rCBF, we believed that a comparison between remitted OCD patients using different drugs (SSRIs or SSRIs plus quetiapine) and healthy controls of the characteristics of the rCBF changes would be potential clinical valuable. The aim of this study was to summarize the characteristics of rCBF before treatment occurring in OCD patients who responded to SSRIs or SSRIs plus quetiapine using the SPECT technique and SPM evaluation to guide the option of clinical pharmacotherapy in OCD patients.

## Materials and methods

### Subjects

We recruited 30 untreated, first-episode patients with OCD based on the DSM-IV criteria (American Psychiatric Association, 1994), Yale-brown Scale (the Yale-brown Obsessive-Compulsive Scale Y-BOCS) total score > 16 points, and HAMD (Hamilton Rating Scale for Depression, 24 item) total score <35. The results of cerebral EEGs were normal. The subjects, who included 18 males and 12 females whose mean age was 27.13 ± 8.98 years, were from in the in/out-patient clinic in the department of psychology at the third hospital of Sun Yat-sen University. Thirty voluntary controls individually matched for age, sex, handedness, level of education and intelligence quotient (IQ), including 17 males and 13 females with a mean age of 29.3 ± 6.8 years, were free of neurological and psychiatric disorders and without any history of OCD. All patients were free of serious body or brain organic disease, substance abuse, and other neurological or psychiatric disorders including depression (HAMD score >35), schizophrenia, bipolar disorder, tics or alcohol/substance abuse. All participants gave written informed consent to participate in the study after the procedures were explained and all recommendations of the local ethical committee (Third Affiliated Hospital Of Sun Yat-sen University) were met. The demographic and clinical data of the OCD patients and healthy controls were shown in Table 1.

**Table 1:** Demographic and clinical data for OCD patients and healthy controls

**Variables**	**Pre-treatment groups (case)**			**Treatment remission groups (case)**		
	**OCD group (19)**	**Controls (30)**	**P**	**SSRI (11)**	**SSRI/quetiapine (8)**	**P**
sex (female, %)	9(47)	13(43)	0.214	5(45)	4(50)	0.305
age (years)	27.21 ± 8.75	29.25 ± 6.81	0.291	28.18 ± 8.2	26.78 ± 7.66	0.286
education (years)	12.66 ± 3.17	13.30 ± 2.47	0.667	12.12 ± 3.77	12.53 ± 3.13	0.797
length of illness (years)	2.47 ± 1.33		—	2.33 ± 1.99	2.97 ± 1.34	0.714
pre-treatment scales						
Y-BOCS total score	24.55 ± 6.72	11.12 ± 5.12	0.013	24.36 ± 6.22	24.55 ± 7.41	0.731
HAMD total score	21.21 ± 4.36	16.33 ± 2.41	0.044	21.30 ± 4.11	21.00 ± 4.17	0.712
Post-treatment scales						
Y-BOCS total score	12.48 ± 5.20 *		—	11.51 ± 5.01 *	11.36 ± 5.14 *	0.512
HAMD total score	17.31 ± 3.49 *		—	17.03 ± 3.18 *	17.21 ± 3.33 *	0.647

*p < 0.01, compared to the pre-treatment score.

### Regional cerebral blood flow (rCBF) imaging protocol

The rCBF imaging was performed using dual-head gamma cameras (SPECT, Discovery VH, GE Healthcare, USA) under resting conditions in quiet surroundings with the subjects’ eyes covered for approximately 30 minutes after each subject was injected with approximately 740 MBq Tc-99 m ethylcysteinate dimer (Beijing atom high-tech Co. Ltd, China). The 64 frames (40s per frame in a matrix of 128 × 128 pixels) were acquired over a least radius orbit with a dual-headed rotating camera equipped with low-energy high-resolution parallel-hole collimators. The raw data were pre-filtered with a Butterworth low pass filter (Order 10, cutoff frequency 0.45 cycles/pixel) to minimize noise and then reconstructed using a filtered back projection algorithm and Chang’s first-order post-processing attenuation correction (0.13/cm as a routine attenuation coefficient in the clinical setting) [3]. The reconstructed data were transformed to DICOM 3.0 format files and exported to a general workstation.

### Pharmacotherapy and assessment instruments

Patients underwent treatment with selective serotonin reuptake inhibitors (SSRIs) with or without the addition of a low dose atypical anti-psychotic. The choice of SSRI was based on the individual sex, age, clinical symptoms, length of illness and side-effect history of each patient, and the patients were scheduled to receive paroxetine, fluvoxamine, sertraline, citalopram and fluoxetine accordingly. Patients were started on a low dose SSRI (50 mg/day for fluvoxamine and sertraline, 20 mg/day for fluoxetine, paroxetine and citalopram). The dosage of SSRIs, if tolerated, was slowly increased to the maximum recommended for OCD. The duration of the trial of treatment was 24 weeks. Y-BOCS and HAMD assessments were performed at weeks zero, two, four, and afterwards, later follow-up assessments were performed. To ensure that clinician-rated measures were consistent across patients and time-points, raters met regularly to review the procedures and to discuss their experiences using the measures.

### The assessment of the clinical remission response and groups

We adopted the following clinical criteria: a >50% reduction in the Y-BOCS total scores for the clinical remission of pharmacotherapy; a reduction of >25% and <50% in the Y-BOCS total scores for the clinical response of pharmacotherapy; and a reduction of <25% in Y-BOCS total scores for the clinical incomplete responses of pharmacotherapy at the end of a 12-week treatment with a high-dose SSRI.

A recommended augmentation dose of quetiapine (200–300 mg/day) was slowly added to the treatment in patients who had incomplete responses to SSRI treatment at the end of 12 weeks of treatment with the maximum tolerated doses. Of the 30 patients, eight dropped out during follow-up visits because of a lack of response to the treatment, and three patients dropped out because of drug side effects. Finally, 19 OCD patients who achieved a clinical remission (eleven patients responded to SSRI treatment and eight patients responded to SSRIs plus quetiapine) were included in the study. The results of this study were based on the data from those 19 patients (ten men, nine women) who completed the study and were divided into SSRIs treatment and SSRIs plus quetiapine treatment groups.

### SPM analysis

All imaging data were converted to the analyzed format using a freeware analysis viewer (MRIcro, version 1.40). All DICOM data were processed with statistical parametric mapping (SPM 5) software (free downloaded from http://www.filion.ucl.ac.uk/spm) running on MATLAB 6.5 (Mathworks Inc., Sherborn, MA). The image data were spatially normalized into a standard stereotactic space as defined by the atlas of Talairach and Tournoux (Talairach and Tournoux, 1988). The normalized images were smoothed with a 10-mm isotropic full-width, half-maximum Gaussian kernel to account for inter-subject differences in anatomy and to allow for valid statistical inference according to Gaussian random field theory.

The rCBF images from the two OCD patient groups and the controls were analyzed using the SPM software and a two samples model. Correlation between the rCBF and Y-BOCS score within the OCD group was performed using a single subject and covariates-only model in SPM analysis. The scores of Y-BOCS were entered as covariates in the statistical analysis, which used a general linear model, of each association. The rCBF image was modelled using a covariance scaling of activity to a normal mean global activity of 50 ml/100 g/min. For the general linear model, the estimated threshold was set to P < 0.001 and a family-wise error (FWE) correction and a small-volume correction (sphere of radius 50 pixels) were used.

### Statistical analysis

Statistical analysis was performed using SPSS version 17.0 for Windows. One-way analysis of variance (ANOVA) and chi-squared statistics were used to determine the statistical significance of age, sex, years of education, length of illness, Y-BOCS and HAMD total score.

## Results

### Demographic and clinical data of OCD patients and healthy controls

OCD patients who completed the study and the control group were not significantly different with respect to sociodemographic characteristics (Table 1). In addition, no significant differences was observed between the OCD patients who completed the study and those who dropped out with respect to age (p = 0.234), gender (p = 0.534) and the length of illness (p = 0.325). The clinical and demographic features of the SSRI and the SSRI plus quetiapine groups were also summarized in Table 1. No significant differences were found between the two groups in terms of other clinical characteristics. A 63.3% (19/30) patients who showed improvement in obsessive-compulsive symptoms (compared to the initial Y-BOCS scores) was observed in all patients at 24 weeks. The Y-BOCS and HAMD scores decreased significantly during the remission period compared to pre-treatment (t = 5.63, p = 0.000; t = 6.11, p = 0.000).

### The rCBF of the SSRI group and normal controls before treatment

**Figure 1:**
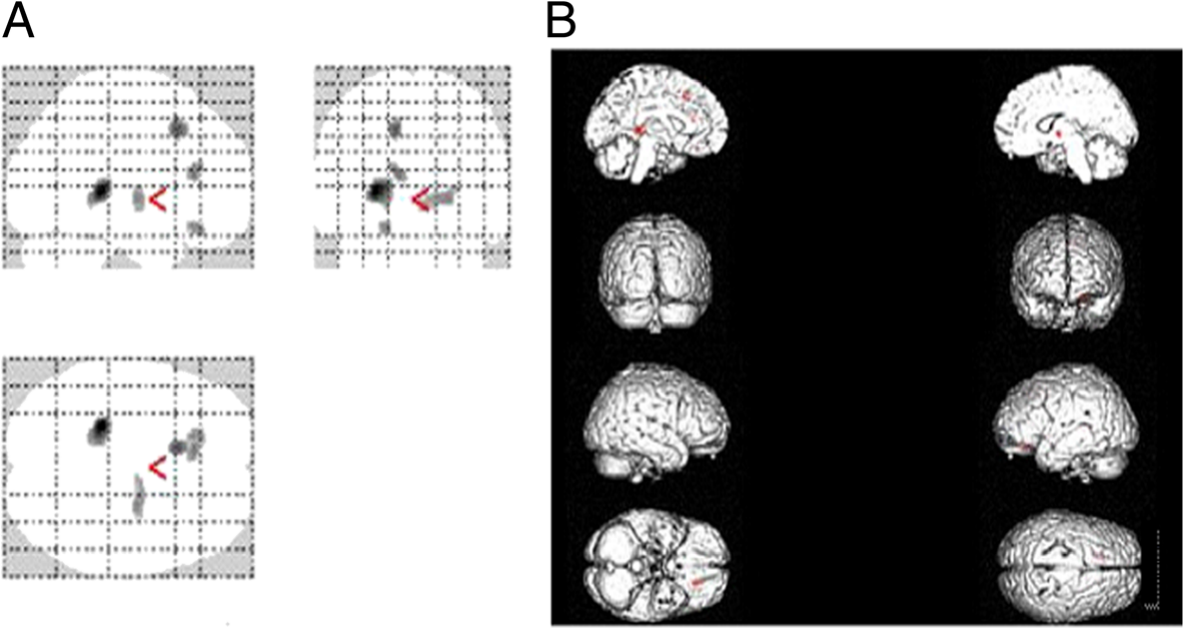
**The image A was the most intensity projection image of the increased rCBF focuses in the patients with SSRI treatment.** The image **B** was the three dimensional image of rendered with increased rCBF focuses.

The increased rCBF was found in SSRI group compared to normal controls before treatment. The increased rCBF regions primarily included the left lenticular nucleus, left thalamic lateral nucleus, left medial frontal gyrus, left anterior cingulate, right globus pallidus and right thalamic lateral nucleus (Figure 1). The coordinates and Z scores of the abnormal cerebral regions were shown in Table 2.

**Table 2:** The coordinates and Z scores of abnormal rCBF in SSRI group and normal controls before treatment

**Hemisphere**	**Brain region**	**Coordinates(mm)**^**a**^			***Z *value**^**b**^	***P *value**
		**X**	**Y**	**Z**		
left	lenticular nucleus	**−16**	6	−8	5.64	0.000
left	Thalamus lateral nucleus	−26	−32	6	4.62	0.000
left	Medial frontal gyrus	−12	20	44	4.31	0.000
left	Anterior cingulate	−10	32	16	4.16	0.000
right	Globus pallidus	24	−8	2	4.13	0.000
right	Thalamus lateral nucleus	12	−6	0	4.03	0.000

^a^ Coordinates from the stereotaxic atlas of Talairach and Tournoux.

^b^ Peak activation in a cluster of at least 50 voxels in which the difference survived a threshold of *P* < 0.001 in a random-effects analysis.

### The rCBF of the SSRI plus quetiapine group and normal controls before treatment

**Figure 2:**
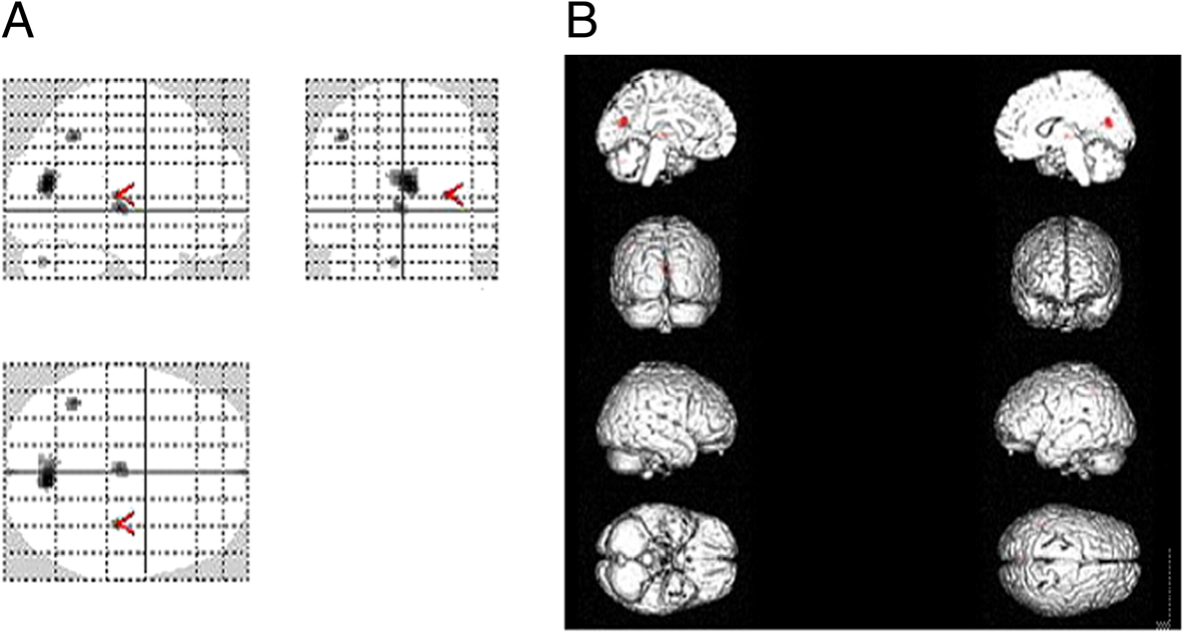
**The image A was the most intensity projection image of the decreased rCBF focuses in the patients with OCD.** The image **B** was the three dimensional image of rendered with decreased rCBF focuses.

**Figure 3:**
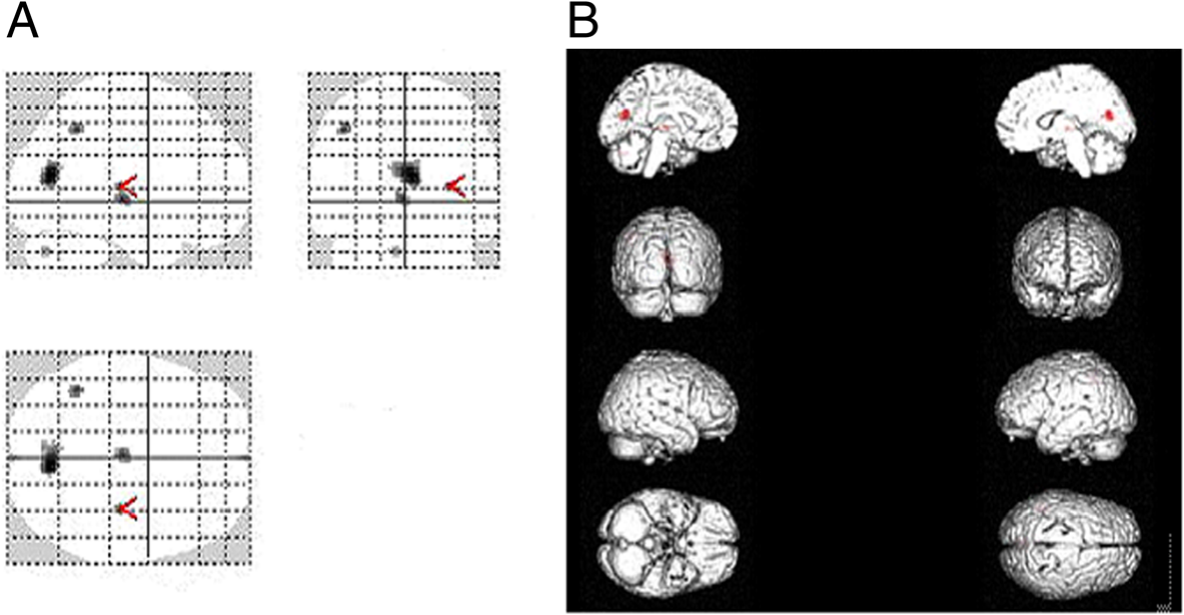
**The image A was the most intensity projection image of the increased rCBF focuses in the patients with OCD.** The image **B** was the three dimensional image of rendered with increased rCBF focuses.

The increased and decreased cerebral perfusion was found in the SSRI plus quetiapine group. The regions of decreased rCBF primarily included the left inferior parietal lobule, left vermis cerebelli and right occipital wedge leaf (Figure 2). The regions of increased rCBF primarily included the left tonsil of the cerebellum, left globus pallidus, right thalamoc lateral nucleus, right lentiform nucleus, and right middle frontal gyrus (Figure 3). The coordinates and Z scores of the abnormal cerebral regions were shown in Table 3.

**Table 3:** The coordinates and Z scores of abnormal rCBF in SSRI plus quetiapine group and normal controls before treatment

**Hemisphere**	**Brain region**	**Coordinates(mm)**^**a**^			***Z *scores**^**b**^	***P *value**
		**X**	**Y**	**Z**		
Decreased rCBF						
left	inferior parietal lobule	−42	−52	46	6.98	0.000
left	Vermis cerebelli	−6	−74	−32	6.91	0.000
right	Occiput wedge leaf	6	−70	16	7.06	0.000
Increased rCBF						
left	Tonsil of cerebellum	−34	−58	−38	5.09	0.000
left	Globus pallidus	−22	−12	4	4.75	0.000
right	Thalamus lateral nucleus	22	−18	8	4.61	0.000
right	Lentiform nucleus	30	−20	8	6.67	0.000
right	Middle frontal gyrus	12	52	16	4.41	0.000

^a^ Coordinates from the stereotaxic atlas of Talairach and Tournoux.

^b^ Peak activation in a cluster of at least 50 voxels in which the difference survived a threshold of *P* < 0.001 in a random-effects analysis.

### Correlation between the pre-treatment rCBF and change in the Y-BOCS score

Correlation between the pre-treatment rCBF and change in the Y-BOCS score Among SSRI group (n = 11), the reduction in the Y-BOCS score was negatively correlated with the pre-treatment rCBF in the left frontal lobe, left cingulate and bilateral thalamus (P < 0.001, FWE-corrected) . Among SSRI plus quetiapine group (n = 8) the reduction in the Y-BOCS score was negatively correlated with the pre-treatment rCBF in the left and right frontal lobe, thalamus and left parietal lobe (P < 0.001, FWE-corrected) and positively correlated with left cerebelli and right occipital lobe (P < 0.001, FWE-corrected). Those results suggest that an increased rCBF in forebrain regions may predict a improvement in OCD severity after SSRI treatment, and that an increased in forebrain regions and a decreased in posterior brain regions rCBF may predict a improvement in OCD severity after SSRI plus quetiapine treatment.

## Discussion

In our study, the increased rCBF was found in the SSRI treatment group compared to normal controls before treatment, and the area of increased rCBF primarily included the left lenticular nucleus, left thalamic lateral nucleus, left medial frontal gyrus, left anterior cingulate, right globus pallidus and right thalamic lateral nucleus. These results suggest that only increased rCBF in forebrain predicts response to SSRIs; similar findings were reported previously in OCD patients [19,20]. Functional imaging studies using SPECT techniques have proposed that a dysfunction in these diffuse cortical networks would result in the emergence and maintenance of repetitive thoughts and characteristic OCD behavior. However, few studies have investigated these brain imaging findings associated with the clinical remission observed following the use of SSRIs or SSRIs plus quetiapine in OCD patients. In a previous SPECT study by Hendler [20], it was found that, after six months of sertraline treatment, OCD patients responding to SSRIs showed increased activation in the frontal cortex during both relaxing and symptom-provoking conditions. The authors proposed that the response to SSRIs may be related to the short-term plasticity of cortical neurons. However, Rauch [21] used SPM methods to investigate predictors of the response to fluvoxamine treatment with contamination-related OCD patients in a PET study in the context of a symptom-provocation paradigm. This study reported that decreased rCBF values in the OFC and increased rCBF values in the posterior cingulate cortex predicted a better response to treatment.

A few studies have reported that posterior brain regions are associated with the response to SSRI treatment. In this study, increased and decreased rCBF was found in the SSRI plus quetiapine group. The regions of decreased rCBF primarily included the left inferior parietal lobule, left vermis cerebelli and right occipital wedge leaf. The regions of increased rCBF primarily included the left tonsil of the cerebellum, left globus pallidus, right thalamic lateral nucleus, right lentiform nucleus, and right middle frontal gyrus. Ho Pian et al. [22] reported that the pre-treatment cerebellar and whole brain HMPAO uptake was higher in subjects that responded to fluvoxamine treatment compared to non-responders. Nabeyama et al. [23] reported that the dysfunction of posterior brain regions, especially the cerebellum, is involved in the pathogenesis of OCD and that a normalization of function can occur with an improvement in OCD symptoms. Therefore, posterior brain regions such as the cerebellum and occipital lobe might be associated with the subsequent treatment response to selective serotonin reuptake inhibitors (SSRI) in patients with OCD. Several reports have suggested that the activities of forebrain and posterior brain regions such as the cerebellum and occipital lobe might be involved in the pathophysiology of OCD [3,12,23]. Our results completely support this conclusion.

Before treatment, SSRI responders had the increased rCBF in forebrain. SSRI plus quetiapine responders had the reduced rCBF in cerebellum, occipital cortex and parietal cortex and increased rCBF in forebrain, which is correlated with the reduction of Y-BOCS scores. SSRI plus qurtiapine responders may be a subgroup representing a reduced capability for establishing neuronal connectivity compared to SSRI responders. Hendler [20] argued that SSRI nonresponsive OCD patients might lack the capability for cortical plasticity by SSRI due to more cortical deficits. Thus these patients may require a more complex modulation of fronto-thalamic-striatal circuits for achieving clinical improvement. Quetiapine augmentation maybe mediated by increasing rCBF in cerebellum, occipital cortex and parietal cortex regions as reported in the patient with psychotic disorders [24,25]. However, this interpretation should be cautiously considered because non-responder patients receiving quetiapine is lacked in our research.

The limitations of the present study include the fact that the low spatial resolution of brain SPECT imaging scintillation cameras (10 mm) limits the detection of abnormalities in small brain regions or the identification of discrete brain abnormalities. In addition, the sample size was small, and thus our results may be difficult to generalize. We require a larger number of subjects to analyze the results with reference to the heterogeneity of OCD and to better investigate the correlations between changes in the rCBF and symptom improvement. Furthermore, we failed to examine the non-responsive patients due to the large dropout rate. A study design requiring a longer follow-up period may have a negative effect on the patients’ compliance to the study protocol.

## Conclusions

To our knowledge, there is few study investigating the brain imaging findings associated with clinical remission in OCD patients. We found that SSRI responders were characterized by increased rCBF in forebrain regions, including the frontal cortex, basal ganglia, thalamus and cingulate gyrus. The SSRI plus quetiapine responders were characterized by increased rCBF changes in the forebrain regions along with decreased rCBF changes in posterior brain regions, including the occipital lobe, inferior parietal lobe and cerebella. Notwithstanding the limitations mentioned above, before treatment the changes in rCBF are associated with pharmacotherapy and clinical improvement in OCD patients and may be valuable in guiding the choice of clinical pharmacotherapy in OCD patients.

## Competing interests

All authors declare that they have no competing interests.

## Authors’ contributions

The work was carried out by collaboration among the authors. SLW conceived and designed the experiment; CMH, CMF and JHY recruited and tested the participants; HW and JHY analyzed the data; SLW wrote the paper. All authors contributed, read, and approved the final manuscript.
